# Preliminary validation of the virtual bariatric endoscopic simulator

**DOI:** 10.1016/j.igie.2024.08.003

**Published:** 2024-09-18

**Authors:** Utku Erden, Mark A. Gromski, Suvranu De, Doga Demirel

**Affiliations:** (1) Department of Computer Science, Florida Polytechnic University, Lakeland, Florida, USA; (2) Division of Gastroenterology, Department of Medicine, Indiana University School of Medicine, Indianapolis, Indiana, USA; (3) College of Engineering, Florida A&M University-Florida State University, Tallahassee, Florida, USA; (4) School of Computer Science, University of Oklahoma, Norman, Oklahoma, USA

## Abstract

**Background and Aims:**

Obesity is a global health concern. Bariatric surgery offers reliably effective and durable weight loss and improvements of other comorbid conditions. However, the accessibility of bariatric surgery remains limited. Minimally invasive techniques, including endoscopic sleeve gastroplasty (ESG), have emerged to bridge this gap. To effectively complete the ESG procedure, one requires skill in multiple complex interventional endoscopic maneuvers. This requisite expertise poses challenges for training in this burgeoning field.

**Methods:**

We designed the virtual bariatric endoscopic (ViBE) simulator software to mimic the ESG procedure accurately. The ViBE simulator features a detailed simulation of an endoscope equipped with an endoscopic suturing system and a high-resolution stomach, enhancing the visualization of procedural details. Furthermore, the simulator incorporates performance metrics using a reverse scoring system to evaluate users’ proficiency in tasks such as argon plasma coagulation (APC) marking, suturing, and cinching. To validate the simulator, we conducted a study involving experts and novices at the Indiana University School of Medicine, where participants engaged with the simulation environment in a series of training tasks.

**Results:**

Twelve participants, comprising 5 experts and 7 novices, were asked to complete a post-training questionnaire featuring 7 items, rating each on a Likert scale. The APC task realism received the highest score, averaging 3.83. The usefulness of improving endoscopic technical skills averaged 3.08, with the realism of cinching the knot and suturing tasks receiving scores of 3.17 and 3.25, respectively, suggesting a generally positive reception. Automated performance metrics indicated that, on average, experts outperformed novices by 10.83 points.

**Conclusions:**

The ViBE simulation strives to replicate the steps of the ESG within a virtual environment. Our primary objective in developing this simulator was to enhance the learning curve for endoscopic suturing and ESG techniques, thereby safely extending these skills to a broader patient base.

Obesity remains a global public health concern.^1^ Individuals falling into obesity class 2 (body mass index, 35 kg/m^2^ to <40 kg/m^2^) or class 3 (>40 kg/m^2^) experience substantial advantages from bariatric surgery, recognized as the most effective approach for achieving sustained weight loss in the long term.^2^ Despite its effectiveness as a weight loss solution, only 1% of eligible patients undergo bariatric surgery because of accessibility, cost, and concerns about associated risks.^3,4^ Consequently, this has led to the exploration of innovative alternatives.

Among these is the emergence of endoscopic sleeve gastroplasty (ESG), a minimally invasive procedure using endoscopic suturing to reduce stomach size and facilitate weight loss,^5^ emulating the mechanism seen in laparoscopic sleeve gastrectomy. This endoluminal, organ-sparing method has proven to be safe and technically viable through various studies, leading to notable reductions in average weight and body mass index, nearly approaching that of sleeve gastrectomy.^6–9^ However, acquiring proficiency in this technique poses inherent difficulties, requiring specialized skills and extensive training, and has a gradual learning process.^10,11^

In response to the imperative need for comprehensive training, virtual simulations became a well-established tool for augmenting medical education and practice.^12^ Hadi and Singh^13^ discussed the complexities of ESG training programs, emphasizing the necessity for technical expertise in endoscopy and suturing. Industry-sponsored and endoscopic society–supported courses on porcine models are standard for gaining endoscopic skill proficiency, in addition to the standard methodical apprentice-type model on human patients, but emerging virtual reality (VR)-based suturing training aims to improve the training paradigm in a risk-free environment. Currently, validated methods for evaluating technical skills in ESG procedures beyond proctor feedback are lacking.

Controlled settings like skills laboratories and simulators provide customized and low-pressure training, enabling practice until proficiency is attained. These simulators provide haptic feedback and a self-paced learning environment. ESG studies indicate that proficiency often requires 29 to 38 procedures for novices and 35 to 53 procedures for experienced endoscopists, with some needing up to 100 cases for mastery. Pretraining with VR significantly reduces the number of procedures needed for proficiency and safety.^14^ Advancements include the study by Lewis et al^15^ on jejunojejunostomy training using a VR simulator, demonstrating evidence based on relationships to other variables across different surgeon expertise levels. Although the VR module lacks concurrent validity with real-life surgery, surgeons found the module useful for training.

Recent advancements in VR robotic surgery simulators, such as improvements in haptic feedback and technical enhancements, have significantly enhanced the field. The latest generation of VR applications features high pixel density displays, improved refresh rates, and better positional tracking, notably benefiting VR robotic surgery simulators like laparoscopy simulation.^16^ For instance, Sankaranarayanan et al^17^ used VBLaST-PT, a virtual simulator for laparoscopic surgery tasks that used hardware like force feedback Phantom devices and interactive simulation technology. Converting this simulator to a Gen2-VR system involves using a VR headset and introducing distractions and interruptions to replicate actual surgical conditions, revealing decreased performance during tasks with added disruptions.

Despite the abundance of virtual simulations for bariatric surgery, simulations for ESG are lacking. The inception of the virtual bariatric endoscopic (ViBE) simulator signified a transformative leap in ESG training methodology.^18^ To the best of our knowledge, the proposed ViBE simulation is the sole educational simulation for ESG training.

## METHODS

The ViBE simulator uses dedicated simulation software, accompanied by user performance metrics, to both facilitate and evaluate its outcomes. Through a user study, we assessed the simulator’s trustworthiness, usefulness, and realism.

The primary aim of this study was to develop and validate the software component of the ViBE simulator through face validation and evidence based on test content and on relationships to other variables. We used a primitive keyboard and mouse input system to confirm the validity of only the software side of the ViBE simulator. This approach was adopted to incorporate feedback into the software system before hardware integration and mitigate any potential bias the simulator might receive with a high-fidelity haptic hardware setup with VR integration. The ViBE simulator replicates the intricate steps involved in ESG, such as endoscopic marking, suturing, and cinching.

To validate the ViBE simulator, we used pretraining and post-training questionnaires, performance metrics, and simulation performance metrics collected from the simulation. Questionnaires assessed the simulation’s usefulness, realism, and trustworthiness. On the other hand, performance and simulation performance metrics were used to compare differences between expert and novice surgeons. Our study used the performance metrics established and validated through real performance videos.^19–21^ These metrics provided automated objective results.

### Simulation software

**Figure 1.**
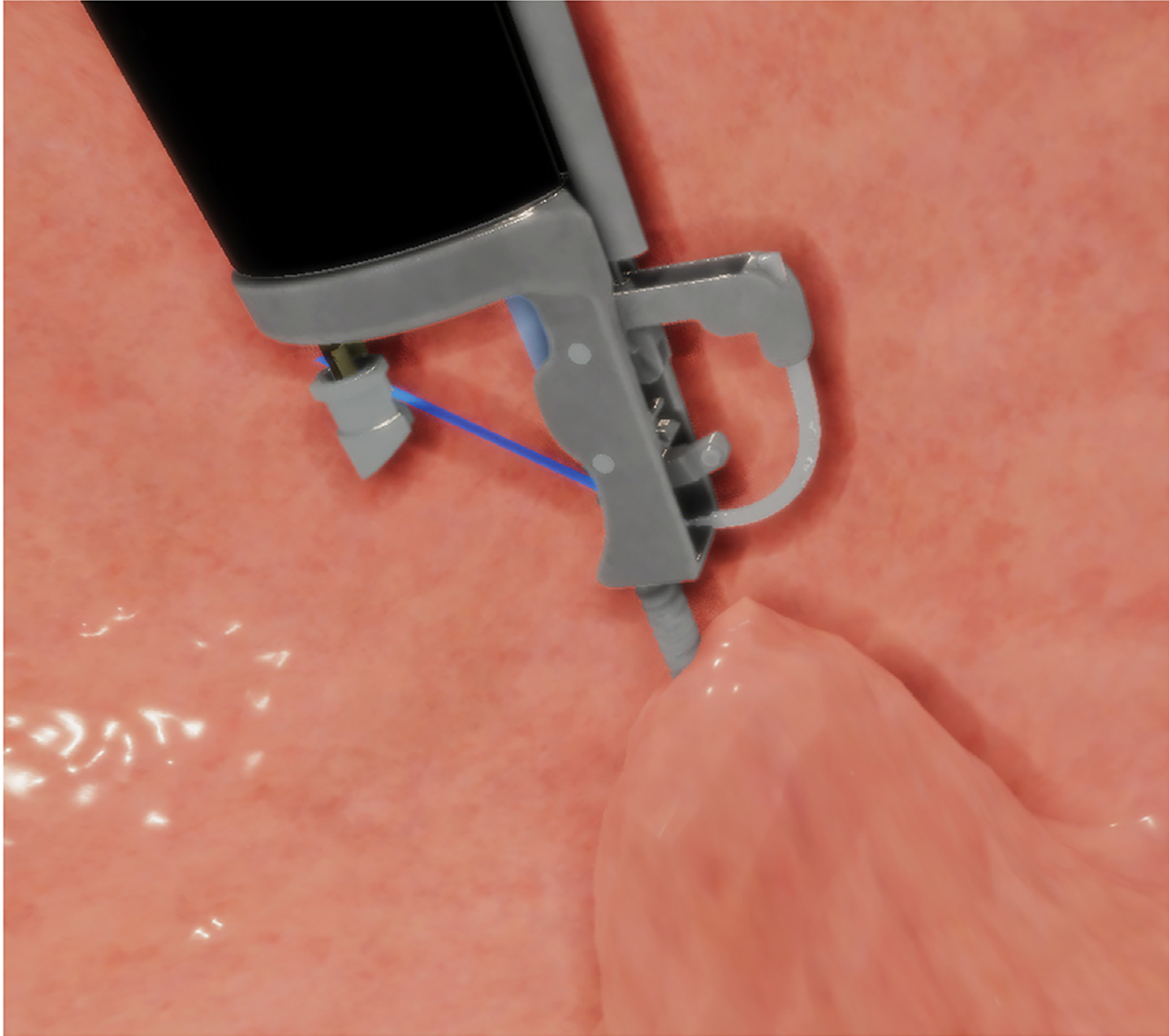
Simulated suturing phase in the virtual bariatric endoscopic simulator.

Two modules provided the ViBE simulator software functionality: simulation and performance metrics. We worked with expert physicians to assemble realistic visuals for the simulation, shaping the surgical environment based on their insights, as seen in Figure 1. The stomach, portrayed with an endoscope and endoscopic suturing system, served as the central element in the simulation.

The simulation software ensures realistic interaction within the endoscopic environment. The software achieves this by numerically computing the physical behavior of simulated organs and facilitating collision detection and response between the stomach and the endoscope with the endoscopic suturing system attachment, enhancing the realism of the simulation. Additionally, we incorporated soft-body behavior into the stomach to enhance the simulation’s realism. To replicate intricate soft-body physics, particularly in simulating the stomach, we implemented extended position-based dynamics.^22^ This approach allowed us to decouple the simulation frequency from object stiffness, enabling the use of a larger number of particles^23^ to accurately sculpt the stomach’s shape. These particles were governed by constraints and finely tuned for soft-body deformation, shaping the mesh using linear skinning techniques.^24^ We integrated shape-matching constraints with oriented particles for the detailed simulation of the stomach as a soft body.^25–27^

**Figure 2.**
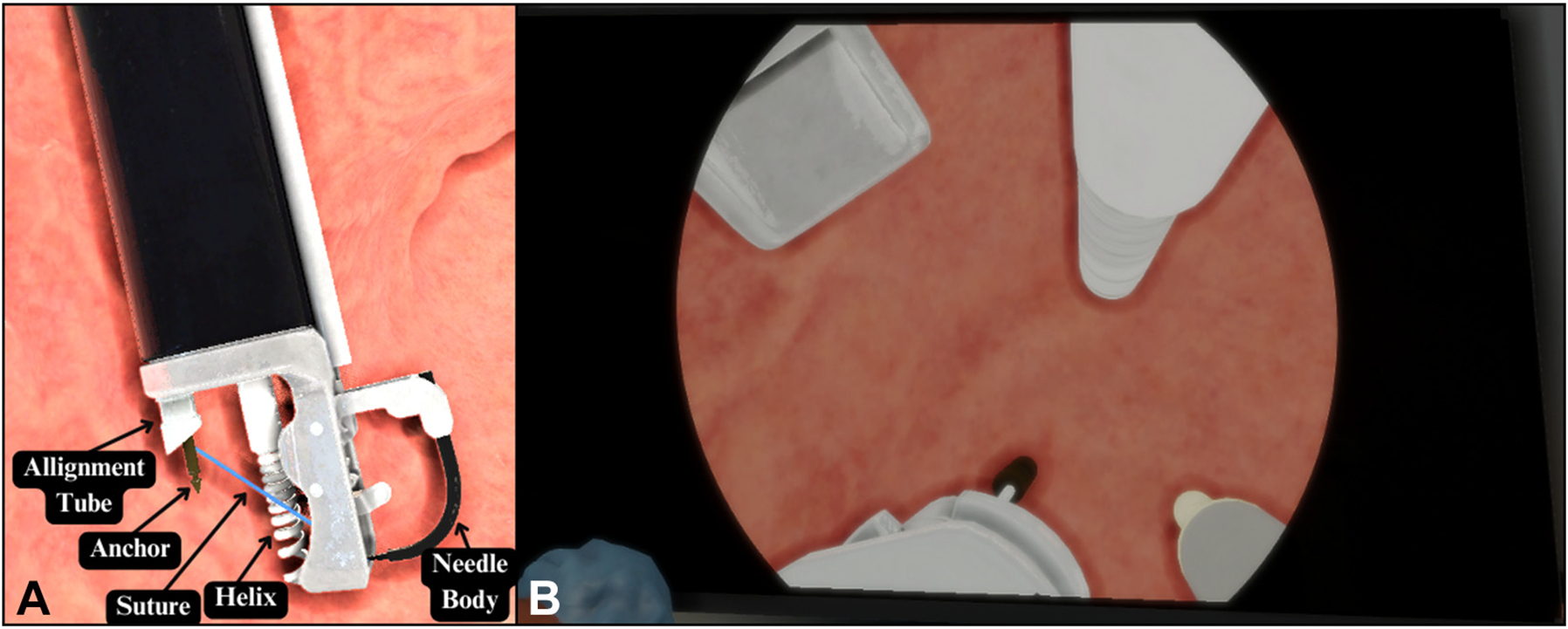
**A,** Endoscopic suturing platform components. **B,** Endoscopic view. *APC*, Argon plasma coagulation.

The endoscope and endoscopic accessories comprise 5 fundamental elements, as depicted in Figure 2A, whereas Figure 2B visually represents the simulated environment. Among these elements, the argon plasma coagulation (APC) probe was created with the primary simulated objective of marking the stomach walls. The helix functionally captures and pulls the stomach tissue into the suturing apparatus to facilitate full-thickness bites. The needle driver and needle components are used to pass the suture material through the target tissue. On completing all suture placements, the cinch is deployed, and the suture set is finalized.

The second component of the simulation software comprises performance metrics, which were used for the evidence based on relationships to other variables of our simulation. We used the performance metrics introduced and validated in our previous work.^19–21^ These metrics are integrated into the simulation to provide automatic and objective feedback on completion. Of 45 metrics,^20^ 8 were related to the insertion and diagnosis of upper endoscopy, 4 pertained to APC marking, 4 were associated with the insertion of the suturing arm, 18 were focused on suturing, 2 were related to adverse events, 1 addressed the aspect of time, and 8 were centered around communication in the procedure room. For this study, we implemented 19 metrics from APC marking, suturing, adverse events, and time completion metrics, because those were our simulation’s main tasks. An inverted scale was used for the performance metrics, with 0 being the best possible score and 5 noting a poor performance. An inverted scoring system was used to integrate time variables more seamlessly into the overall scoring process.^20^ Performance metrics included in the simulation can be seen in Table 1.

### Experimental design

At Indiana University School of Medicine, a user study with 12 participants was segmented into distinct groups based on their expertise (Indiana University School of Medicine Institutional Review Board Protocol no. 18374). Participants were initially classified into 2 proficiency levels: experts (n = 5) and novices (n = 7). Expertise classification relied on specific criteria, considering both the duration of tenure in their positions and the cumulative count of endoscopic procedures performed. Individuals with >5 years of endoscopy experience, >1500 total procedures, and >10 endoscopic suturing procedures were categorized as experts, as seen in Table 2.

Each participant received both pretraining and post-training questionnaires. The pretraining questionnaire encompassed inquiries regarding anonymous demographic details (age, sex), role within the medical field, tenure in their current role, aggregate count of endoscopy procedures conducted in the last 6 months and throughout their career, total experience in endoscopic suturing procedures, and count of ESG procedures in their career. In the post-training questionnaire, participants were tasked with assessing various aspects of the simulation’s realism, including its fidelity concerning APC marking, suturing, cinching, and overall simulation experience, as seen in Tables 3 and 4. Additionally, participants were asked to evaluate the simulation’s effectiveness in enhancing hand–eye coordination skills, its overall utility in improving endoscopic technical proficiency, and perceived reliability of the simulation in quantifying accurate performance metrics. Ratings were requested on a Likert scale ranging from 1 (the lowest) to 5 (the highest) to understand the perceived quality across these parameters.

## RESULTS

### Post-training questionnaire

**Figure 3.**
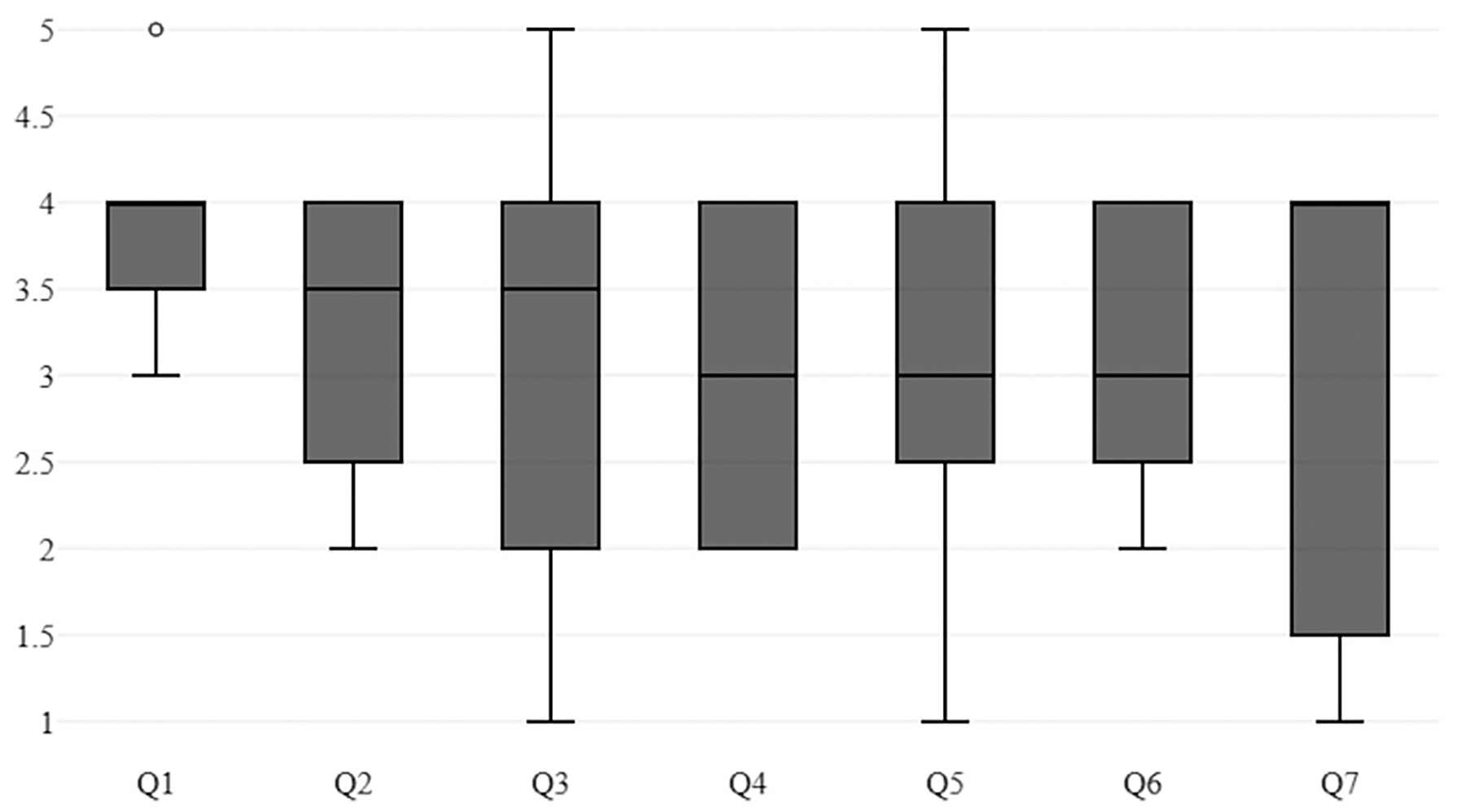
Boxplots of responses from the post-training questionnaire. The first 4 questions correspond to items from Table 3 and the last 3 from Table 4.

For the face validation and evidence based on test content, we used the post-training questionnaire mentioned above. The post-training questionnaire results indicated that every question’s average result was above 3.00. The realism of the APC task was graded as the highest, with 3.83 among the questions. The other tasks, suturing and cinching, received an average grade of 3.25 and 3.17, respectively. The usefulness of improving endoscopic skills was graded as 3.08, whereas the overall simulation realism, usefulness of hand–eye coordination, and trustworthiness of the simulation all obtained the same score of 3.00. Figure 3 displays the boxplots for the post-training questionnaire results.

### Performance metrics

**Figure 4.**
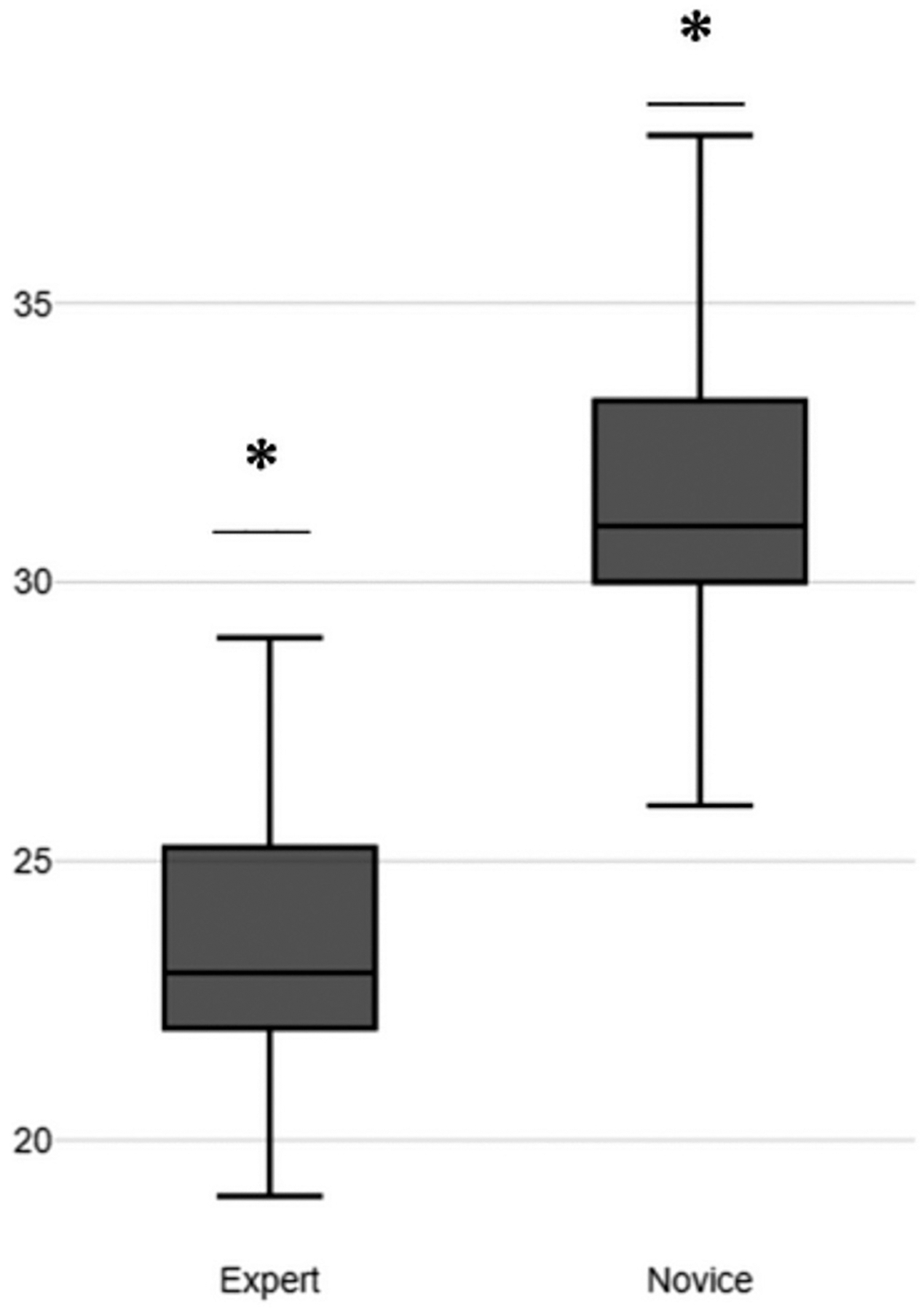
Total score boxplots for novice and expert groups. *Significant difference, *P* < .005.

For evidence based on relationships to other variables, we used our previously defined performance metrics. In these metrics, a score of 0 represents the highest rating and a score of 5 the lowest rating. Expert endoscopists outperformed novice endoscopists in 12 of 19 performance metrics. Experts and novices had the same mean scores of 0 in 4 metrics. Novices outperformed expert surgeons in only 3 metrics: M1, anterior wall marking (expert mean, 2; novice mean, 1.86); M14a, U-shaped pattern (expert mean, 4; novice mean, 3.58); and M18, time completion (expert mean, 6; novice mean, 3.86). For the mean total score, experts (mean, 24.00) outperformed novices (mean, 35.00) by 11 points, as seen in Figure 4. The results of all performance metrics and the total score can be seen in Table 5. Finally, we conducted statistical comparisons between the scores of novice and expert participants using Welch’s *t* test. Our analysis revealed a significant *t* value of −4.0391 (*P* = .0029), indicating statistical significance.

### Simulation performance metrics

**Figure 5.**
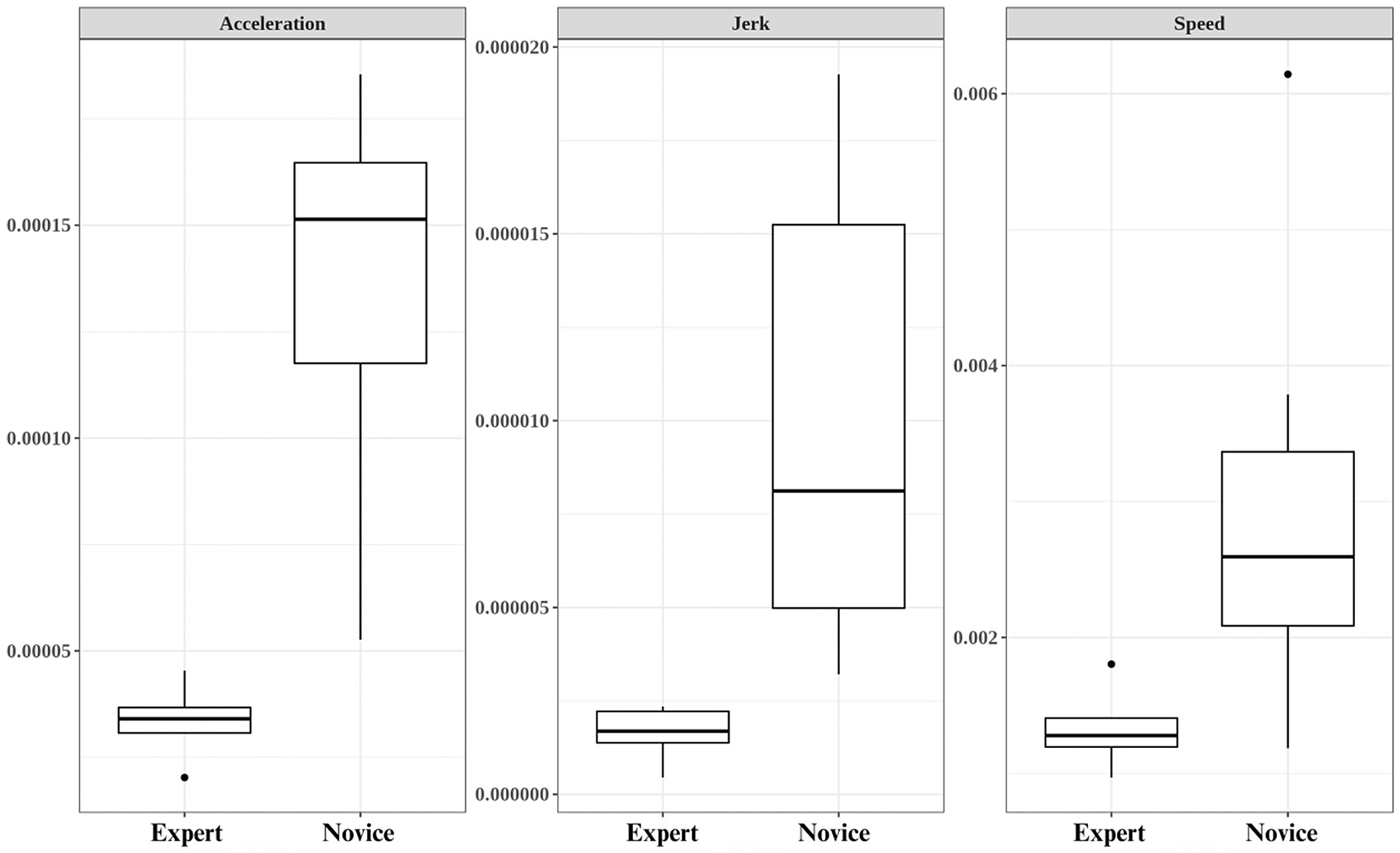
**A,** Acceleration difference between novice and expert groups during suturing. **B,** Jerk difference between novice and expert groups during suturing. **C,** Speed difference between novice and expert groups during suturing.

Finally, we analyzed the data from the simulation and calculated the speed, acceleration, and jerk for all participants, as seen in Figure 5. We performed statistical comparisons between novice and expert surgeons using Welch’s *t* test, as seen in Table 6. Our results displayed significant *t* values and *P* values showing statistical significance of −4.0827 (*P* = .000906), −4.0982 (*P* = .000882), and −4.0988 (*P* = .000881) for speed, acceleration, and jerk, respectively. Lower speed, acceleration, and jerk values indicate smoother and more controlled motion.

## DISCUSSION

The outcomes derived from the post-training questionnaire offer insight into participants’ perceptions of tasks of the simulation. The high average score of 3.83 for the realism of APC indicates that participants found this task convincingly realistic. Moreover, enhancing endoscopic skills scored the highest among the utility metrics, with an average rating of 3.08, highlighting the simulation’s perceived value in improving these skills. The similar average scores for the overall simulation realism, usefulness of hand–eye coordination, and trustworthiness of the simulation (all at 3.00) indicate a perceived association between the simulation’s realism, its effectiveness in improving relevant skills, and participants’ confidence in its reliability. These outcomes suggest that the simulation is viewed as a beneficial tool for advancing endoscopic skills.

For the performance metrics, insertion and diagnostic upper endoscopy, insertion of the suturing arm, and reinforcement suture metrics were not included in the simulation because of a lack of specialized hardware. These metric items will be included in the simulation for the complete validation of the ViBE simulator with the specialized haptic hardware for the simulation.

The performance metric results indicate a clear difference between expert and novice users. Experts exhibited an average score of 24.00, whereas novices scored 35.00 out of 104. In 16 of 19 metrics, experts either matched or outperformed novices. The 3 metrics in which novices performed better were M1, M14a, and M18. M18 was calculated using the time of completion data. The average completion time for expert surgeons was 839.8 seconds and for novices, 719 seconds. This shows that expert surgeons prioritized precision and accuracy over speed, taking more time to ensure each procedure step was performed correctly.

On the other hand, all experts completed a mitigation procedure more rapidly in the presence of severe bleeding. Experts used early cinching at 36.46 seconds after severe bleeding; for novices this was 48.75 seconds. Finally, in M8, M12, M15, and M16, both novices and experts achieved a score of 0, indicating that every participant in both groups fulfilled the requirements in the metrics.

The simulation performance metrics showed significant differences between novice and expert surgeons regarding their speed, acceleration, and jerk metrics during suturing. Negative *t* values indicate that experts achieved lower mean scores in these metrics than novices, suggesting a more controlled and smoother suturing motion coupled with consistent speed. The statistical significance of these differences is confirmed by *P* values (speed, *P* = .00090; acceleration, *P* = .00088; jerk, *P* = .00088). These findings support the evidence based on relationships to other variables of this simulator.

Consistent degrees of freedom and critical values across metrics indicate a uniform experimental design and analysis. The experimental conditions and statistical procedures were consistently applied across different metrics, reinforcing the confidence in the statistical significance of the observed differences in speed, acceleration, and jerk metrics between novice and expert surgeons during suturing. The greater variability in performance among novices points to the substantial potential for skill enhancement through focused training, offering valuable insights for surgical training programs to prioritize the development of fine motor skills and narrow the proficiency gap characteristic of less-experienced surgeons. The implications of these differences may indicate that novice endoscopists could benefit from additional training to refine their suturing strategies more in line with the experienced endoscopists.

In conclusion, in this study, we carried out preliminary face validation and evidence based on test content and on relationships to other variables of our ViBE simulation software, which shows promise in improving the training paradigm for the ESG procedure, ultimately aiming to enhance accessibility to this emerging weight loss procedure.

We used pre-training and post-training questionnaires, performance, and simulation metrics throughout the validation process. Post-training questionnaire results were used for the face validation and evidence based on test content of our simulation. We implemented automated, objective, inverted performance metrics for evidence based on relationships to other variables and noted differences between expert and novice performance. Of 19 performance metrics, experts outperformed novices in 12 metric items. Experts and novices had matching grades on 4 metric items, whereas novices only outperformed experts in 3. For the total score, the novices’ average score was 35.43, whereas the experts’ average score was 24.6.

With this work, we established the foundation of an ESG simulation. The ViBE simulator facilitates APC marking, suturing, and cinching tasks of the ESG procedure. It provides automated and objective feedback at the end of the simulation session, enabling participants to refine their skills and expedite their learning.

We continue to incorporate user feedback to enhance the simulator’s performance and plan to integrate a realistic hardware interface in the next development phase. Currently, the communication part is excluded from the scoring system because of its dependence on an assistant. Future work will include a fully capable artificial intelligence assistant that will respond to verbal commands from the surgeon, improving these aspects of the scoring system.

## Figures and Tables

**TABLE 1.** Performance metrics

Metric no.	Metric	Score
*Argon plasma coagulation marking*		
M1	Mark anterior wall (optional)	
	Parallel line	0
	Nonparallel line	3
	No mark	5
M2	Mark posterior wall (optional)	
	Parallel line	0
	Nonparallel line	3
	No mark	5
M3	Mark greater curvature (optional)	
	Parallel line	0
	Nonparallel line	3
	No mark	5
*Suturing*		
M4	Start of suture	
	Start proximal to incisura angularis on the anterior gastric wall	0
	Start at a different location	5
M5	Grasp tissue on anterior wall	
	Grasp near marked tissue (within .5 cm of marking)	0
	Grasp away from the marked tissue	5
M6	Suture the anterior wall	
	Correctly complete suture and anchor exchange	0
	Incorrectly complete suture and anchor exchange	5
M7	Grasp tissue on the greater curvature	
	Grasp near marked tissue (within .5 cm of marking)	0
	Grasp away from marked tissue	5
M8	Suture the greater curvature	
	Correctly complete suture and anchor exchange	0
	Incorrectly complete suture and anchor exchange	5
M9	Grasp tissue on the posterior wall	
	Grasp near marked tissue (within .5 cm of marking)	0
	Grasp away from marked tissue	5
M10	Suture the posterior wall	
	Correctly complete suture and anchor exchange	0
	Incorrectly complete suture and anchor exchange	5
M11	Suture direction (after each anterior/greater curve/posterior suture series)	
	Distal to proximal 1–2 cm	0
	Any other direction/amount	5
M12	Suture bite (per bite)	
	Full thickness	0
	Any other bite	5
M13	Bite amounts per suture set	
	6 or >6 bites	0
	<6	5
M14a	U-shaped pattern (if using U-shaped pattern)	
	Yes	0
	No	5
M14b	Z-shaped pattern (if using a Z-shaped pattern)	
	Yes	0
	No	5
M15	Tighten sutures	
	Release T tag correctly to form a plication using cinching device	0
	Do not release T tag	5
M16	End suture	
	Do not suture fundus	0
	Suture within fundus	5
*Adverse events*		
M17	Severe bleeding	
	Premature cinch (to stop bleeding)	0
	No premature cinch	5
*Time completion*		
M18	Total time	
	First quartile	0
	Second quartile	3
	Third quartile	6
	Fourth quartile	9

**TABLE 2.** Characteristics of experts and novices

Participant no.	Classification	Age (y)	Sex	Hand dominance	Experience	No. of endoscopy cases	No. of endoscopic suturing cases
1	Novice	31	Female	Right	2 y 4 mo	1000	0
2	Expert	42	Female	Right	10 y 5 mo	13,000	10
3	Expert	39	Male	Right	5 y 3 mo	7500	120
4	Novice	36	Male	Left	3 mo	1800	10
5	Expert	43	Male	Right	9 y	9000	30
6	Novice	33	Female	Right	1 y 5 mo	500	0
7	Novice	32	Male	Right	2 y 4 mo	1000	1
8	Expert	48	Male	Right	8 y	18,000	1000
9	Novice	32	Female	Right	3 y 5 mo	1000	0
10	Expert	40	Female	Right	7 y 3 mo	1500	10
11	Novice	29	Male	Right	1 y	70	0
12	Novice	30	Male	Right	1 y 5 mo	40	0

**TABLE 3.** Face validation post-training questionnaire

	Question
Q1	Rate the degree of realism of argon plasma coagulation marking (how realistic it looks) in the simulation
Q2	Rate the degree of realism of suturing (how realistic it looks) in the simulation
Q3	Rate the degree of realism of cinching in the simulation
Q4	Rate the degree of overall realism of the simulation

**TABLE 4.** Evidence based on test content post-training questionnaire

	Question
Q1	Rate the usefulness of the simulation in learning hand–eye coordination skills
Q2	Rate the degree of overall usefulness for improving endoscopic technical skills
Q3	Rate your assessment of how trustworthy the simulation is to quantify accurate measures of performance

**TABLE 5.** Performance metric results

Metric no.	Expert mean	Novice mean
M1	2.00	1.86
M2	1.00	2.86
M3	.00	.71
M4	2.00	2.86
M5	1.00	2.14
M6	2.00	4.29
M7	.00	1.43
M8	.00	.00
M9	.00	1.43
M10	.00	.71
M11	.00	.71
M12	.00	.00
M13	3.00	3.57
M14a	4.00	3.58
M14b	3.00	4.30
M15	.00	.00
M16	.00	.00
M17	.00	.71
M18	6.00	3.86
Total	24.00	35.00

**TABLE 6.** Welch’s *t* test scores comparing novice and expert participants for the suturing task

Values	Speed	Acceleration	Jerk
*t* value	−4.0827	−4.0982	−4.0988
Degrees of freedom	11	11	11
Critical value	1.796	1.796	1.796
*p* value	.00090	.00088	.00088

## Abbreviations:

APC: argon plasma coagulation
ESG: endoscopic sleeve gastroplasty
ViBE: virtual bariatric endoscopic
VR: virtual reality
